# Clinical Outcomes and Prognostic Factors of 695 Nasopharyngeal Carcinoma Patients Treated with Intensity-Modulated Radiotherapy

**DOI:** 10.1155/2014/814948

**Published:** 2014-08-05

**Authors:** Weidong Wang, Mei Feng, Zixuan Fan, Jie Li, Jinyi Lang

**Affiliations:** Department of Radiation Oncology, Sichuan Cancer Hospital, Renmin South Road 4th Section, No. 55, Chengdu 610041, China

## Abstract

*Objective*. The 5-year clinical outcomes and prognostic factors of nasopharyngeal carcinoma (NPC) patients treated with intensity modulated radiotherapy (IMRT) were evaluated. *Methods*. Six hundred ninety five NPC patients primarily treated with IMRT in Sichuan Cancer Hospital from January, 2003 to December, 2006 were analyzed retrospectively, including 540 males and 155 females. The prescription dose was delivered as follows: gross target volume (GTVnx) 67–76 Gy in 30–33 fractions, positive neck lymph nodes (GTVln-R/L) 60–70 Gy in 30–33 fractions, high-risk clinical target volume (CTV1) 60–66 Gy, low-risk clinical target volume (CTV2) 54–60 Gy, and clinical target volume of cervical lymph node regions (CTVln) 50–55 Gy. *Results*. The 5-year local control (LC), regional control, distant metastasis-free survival (DMFS), disease free survival, disease specific survival, and overall survival (OS) rates were 89.8%, 95.2%, 74.1%, 69.6%, 83.2%, and 77.1%. The 5-year DMFS of IMRT and IMRT combined with chemotherapy was 62.1% and 70.9%, the OS of them was 72.9% and 79.1%. The incidence of grade 3 acute and late toxicity was 38.3% and 4.2%, respectively. *Conclusion*. The 5-year LC and OS rate of NPC treated with IMRT was 89.8% and 77.1%. The clinical stage, N stage, volume of GTVnx, and chemotherapy were the main prognostic factor for the OS. Distant metastasis was the main pattern of failure.

## 1. Introduction

Nasopharyngeal carcinoma (NPC) is common among Asians, especially the Southern Chinese, with an incidence rate as high as 30–50 cases/100,000 people. Most nasopharyngeal carcinomas are poorly differentiated and characterized by invasive growth into surrounding tissues and frequent metastasis. In addition, the nasopharynx is adjacent to many important tissues and organs, significantly limiting the feasibility of surgical treatments. However, nasopharyngeal carcinomas are highly sensitive to radiation; therefore, radiotherapy has become one of the primary therapeutic approaches. Intensity-modulated radiotherapy (IMRT) is a major breakthrough in the treatment of nasopharyngeal carcinoma (NPC). It is capable of producing highly conformal dose distributions with steep dose gradients and complex isodose surfaces. Its technical advantages over two-dimensional and three-dimensional conformal radiotherapy have been shown clearly in a number of dosimetric studies [1–4]. Encouraging results with IMRT studies have been reported, and more than 85% locoregional control has been consistently shown [5–12]. Sultanem et al. [13] at the University of California, San Francisco (UCSF), reported the first results of IMRT treatments in 35 cases of nasopharyngeal carcinoma, reporting a 4-year overall survival (OS) rate of 94% and a distant metastasis-free survival (DMFS) rate of 57%. Zhao et al. [14] have reported the results of IMRT treatment in 139 cases of NPC at the Sun Yat-Sen Cancer Center; the 3-year locoregional progression-free survival (LPFS), DMFS, and OS rates were 94.5%, 91.0%, and 86.6%, respectively. Most of the patients exhibited only grades 1-2 acute radiation reactions and grades 0-1 late damage; none of the patients exhibited grade 4 acute reactions or late damage. Taken together, these reports have shown that IMRT technology has incomparable advantages. However, data from large-scale, long-term follow-up studies are still required because of the relatively small number of cases and short follow-up times reported by these published studies. Therefore, we performed a retrospective analysis of the long-term therapeutic effects and the correlated prognostic factors for 695 nasopharyngeal carcinoma patients who were admitted for initial treatment and received full-course IMRT between January 2003 and December 2006 at the Sichuan Cancer Hospital.

## 2. Materials and Methods

### 2.1. Patient Selection

A total of 1800 patients with pathologically confirmed and untreated nasopharyngeal carcinoma were admitted to the Sichuan Cancer Hospital and 763 patients received full-course IMRT between January 2003 and December 2006. 695 cases had complete medical information and entered the retrospective analysis finally. All patients underwent complete physical examination, endoscopy, magnetic resonance imaging (MRI) of the head and neck, chest radiography, and dental assessment. There were 540 males and 155 females. The age of patients ranged from 14 to 74 years, with a median age of 45. According to the pathological classifications of the World Health Organization (WHO) [15], there were 40 cases of type 1 nasopharyngeal carcinoma and 655 cases of type 2 nasopharyngeal carcinoma. According to the UICC2002 staging system, there were 43 cases of stage I, 172 cases of stage II, 268 cases of stage III, and 212 cases of stage IV nasopharyngeal carcinoma (see Table 1).

### 2.2. Radiotherapy

**Figure 1:**
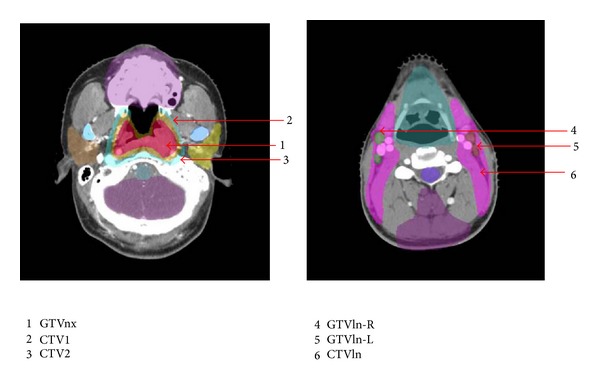
Schematic diagram showing outlines of the target volume.

All of the patients were treated with radical external radiation therapy. The target volumes of the NPC and upper neck were irradiated with full-course IMRT. The patients assumed the supine position on a MedTec positioning system with head, neck, and shoulders fixed and with a 2.0 cm cork in their mouth. Each patient underwent a localized, contrast-enhanced CT scan, with the cranial vertex as the upper limit and 2 cm below the inferior margin of the clavicle head as the lower limit. The scanning layer thickness was 3.0 mm, and the layer interval was 2.5 mm. Registration of diagnostic MRI with planning CT images was performed for all patients for accurate delineation of tumor volumes and critical structures. According to the definitions of the ICRU50 and 62 (International Commission on Radiation Units and Measurements), the target volumes were outlined in each layer of the CT images on an IMRT workstation. The gross tumor volumes of the nasopharynx (GTVnx) and positive neck lymph nodes (GTVln-R/L) were outlined based on the borders of the primary nasopharyngeal tumor and involved lymph nodes as shown by clinical evaluation, endoscopy, CT, and MRI. For patients given induction chemotherapy, the targets are based on the prechemotherapy extent as shown in the MRI images. Clinical target volume (CTV1) includes the GTVnx with a 5 to 10 mm margin, the whole nasopharynx. A smaller margin will be used for the primary tumor where it is adjacent to a critical neurologic structure. Clinical target volume (CTV2) covers the CTV1 and high-risk local structures (including the parapharyngeal spaces, posterior third of nasal cavities and maxillary sinuses, pterygoid processes, base of skull, lower half of sphenoid sinus, anterior half of clivus, and petrous tips). CTVln covers lymphatic drainage regions (including the bilateral retropharyngeal nodes, levels II, III, IV, VA, and VB) (Figure 1). Selective sparing of level IB is considered in N0 disease.

The radiotherapy planning was designed and optimized using the CORVUS 3.4–4.2 inverse treatment planning system. The planning target volume (PTV) was directly generated by the planning system, taking into account any uncertain factors. The specific outward expansion of the boundaries was adjusted according to the structural characteristics of the surrounding tissues. The outlines of CTV1 and CTV2 were reduced to the GTVnx and to 2-3 mm outside CTV1, respectively, when the brain stem and spinal cord were approached. The prescribed doses of each target area were as follows: 66–76 Gy for GTVnx, 60–70 Gy for GTVlnR/L, 60–66 Gy for CTV1, 55–60 Gy for CTV2, and 50–55 Gy for CTVln, each divided into 30–33 deliveries. The dose limits for each normal organ were set according to the Radiation Therapy Oncology Group protocol 0225 (RTOG0225). The target volume and the dosage distribution in the organs at risk were estimated on each CT imaging layer along with the dose-volume histograms (DVH). The target volume dose requires at least 95% of the prescribed dose (V95 ≥ 95%), and the maximum dose of the treatment plan should be inside the target volume. For serial organs, the radiation dose received by 1% of the organ volume should be lower than the maximum tolerance dose of the organ. For parallel organs, the radiation dose received by 33% of the organ volume should be lower than the maximum tolerance dose of the organ. The IMRT plan was implemented through dynamic intensity-modulated coplanar arc irradiation using a multileaf collimator (Nomos mimic). All treatment plans were confirmed by physicians, followed by dosimetric verification and subsequent implementation of the therapy. All of the patients received radiation in the lymph node drainage areas in the lower neck using ^60^Co split-field techniques or 6MV X-ray split-beam techniques with the prescription dose of 50 Gy.

### 2.3. Chemotherapy

Of the 695 patients, 236 received radiotherapy only. Of the 480 patients with stages III-IVB carcinomas, 21 received radiotherapy only (major medical comorbidities precluding chemotherapy) and 459 patients received IMRT combined with chemotherapy. Male and female patients were distributed evenly in the IMRT and IMRT plus chemotherapy cohorts. Of these 459 patients, 52 received induction chemotherapy (100 mg/m^2^ of cisplatin on day 1 and 1,000 mg/m^2^ of 5-FU on days 1 through 5 for 1-2 cycles every 3 weeks) with concurrent chemotherapy consisting of 80–100 mg/m^2^ of cisplatin every 3 weeks for 2 to 3 cycles, whereas 181 received concurrent-adjuvant chemotherapy consisting of 80–100 mg/m^2^ of cisplatin every 3 weeks for 2 to 3 cycles followed by adjuvant chemotherapy 80 mg/m^2^ of cisplatin on day 1 and 1,000 mg/m^2^ of 5-FU on days 1 through 4 for 3 cycles every 4 weeks. 190 patients received concurrent chemotherapy only.

### 2.4. Follow-Up

During the treatment period, all of the patients received weekly physical examinations and blood tests. Acute radiation toxicity was assessed on a weekly basis during radiotherapy in the following domains, according to the Common Toxicity Criteria (version 2.0): radiation dermatitis, mucositis due to radiation, and dysphagia related to radiation and salivary gland changes. Each patient was assessed at regular intervals for treatment response and toxicity after radiotherapy (every 2-3 months during the first 2 years and then every 3-4 months during years 3–5). For each follow-up, routine blood test, biochemistry tests, nasopharyngeal CT or MRI scans, chest X-rays or CT scans, abdominal color Doppler sonography, and isotope bone scans were performed. Late toxic reactions were assessed according to the Radiation Therapy Oncology Group (RTOG) criteria. The follow-up period ended in October 1, 2011. The median follow-up time was 66.4 months (range 7–106 months), with a follow-up rate of 98.1%.

### 2.5. Statistical Analysis

Statistical analysis was carried out using SPSS 13.0 software, and the survival rate was calculated using the Kaplan-Meier method. The follow-up period started from the date of diagnosis and ended on either the date of death or the date of the last follow-up. Acute and chronic adverse reactions were assessed according to the radiation injury standards of the RTOG. Late adverse reactions refer to those that occurred 3 months after the radiotherapy or lasted for over 90 days. The log-rank test was used for univariate analyses, and the Cox proportional hazard model was used for multivariate prognostic analyses. *P* values of less than 0.05 were considered to represent statistical significance.

## 3. Results

### 3.1. Dose-Volume Analysis

The DVH and dose curves were statistically analyzed for all patients. Tables 2 and 3 summarize the dosimetric data of target volumes and selected organs at risk, respectively. As shown, the dose constraints of neurologic organs at risk could all be achieved. The average brainstem and spinal cord *D*_01_ were 49.5 Gy and 37.4 Gy, respectively, which were much lower than those achieved with conventional 2D-RT. The doses for the left and right parotid glands and the left and right temporomandibular joints (*D*_33_) were 31.73 Gy, 32.12 Gy, 40.41 Gy, and 39.12 Gy, respectively (Table 3). For the vast majority of patients, the prescribed dose was delivered to at least 95% of their GTV. The average doses for GTVnx, GTVln-L/R, CTV1, CTV2, and CTVln were 72.6 Gy, 68 Gy, 66 Gy, 58 Gy, and 53 Gy, respectively (Table 2). In general, the dose coverage and uniformity by IMRT were excellent, although for some T3 and T4 patients underdosing a small part of target volumes was inevitable because of the close proximity of critical neurologic structures.

### 3.2. Treatment Outcomes

#### 3.2.1. Survival Results

For all the patients, the 5-year rates of LC (local control), RC (regional control), DMFS (distant metastasis-free survival), DFS (disease-free survival), DSS (disease specific survival), and OS (overall survival) were 89.8%, 95.2%, 74.1%, 69.6%, 83.2%, and 77.1%, respectively. The treatment failed in 126 cases, with a median failure time of 33 months (range 5–101 months); the failed cases included 29 cases (18.2%) of local failure, 13 cases (8.2%) of regional failure, and 117 cases (73.6%) of distant metastasis. There were 133 deaths, including 19 deaths from locoregional relapse (4 patients died of massive nasopharyngeal hemorrhages), 1 death from a fistula hemorrhage due to invasion into the carotid artery, 1 death from rectal cancer, 1 death from liver cancer, and 95 deaths from distant metastasis. The organs affected by distant metastasis were bone (47 cases), liver (26 cases), lung (9 cases), or other organs (13 cases).

#### 3.2.2. Comparison of the Survival Results of IMRT Only and IMRT with Chemotherapy

**Figure 2:**
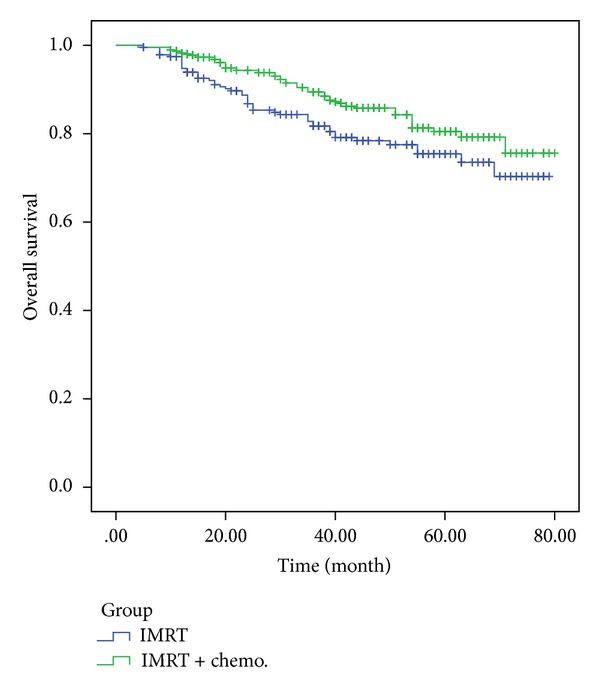
Comparative analysis of the 5-year OS of patients who received IMRT alone and patients who received combined chemoradiotherapy.

**Figure 3:**
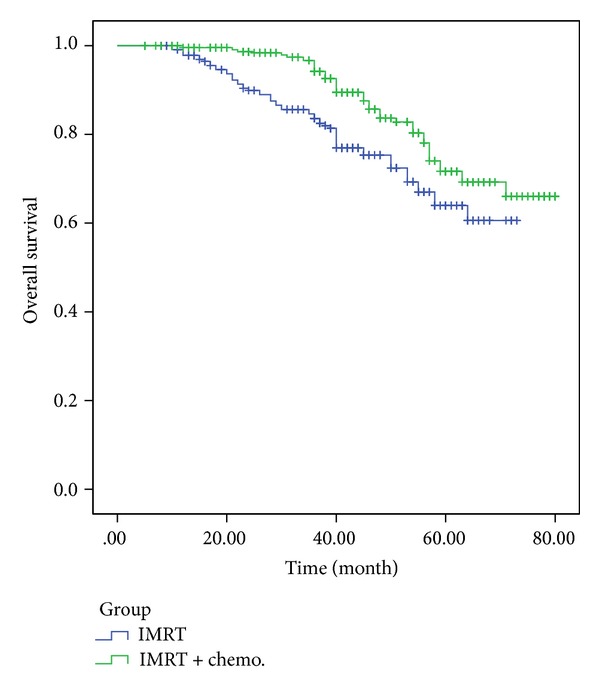
Comparative analysis of the 5-year DMFS of patients who received IMRT alone and patients who received combined chemoradiotherapy.

Of the 480 patients with stage III/IV carcinomas, 21 received IMRT alone, and 459 received IMRT combined with chemotherapy. The 5-year distant metastasis-free survival and 5-year overall survival rate were 62.1% and 72.9%, respectively, for the IMRT-only treatment group and 70.9% and 79.1%, respectively, for the combined chemoradiotherapy group. The 5-year DMFS and OS survival curves of the two groups exhibited statistically significant differences according to the log-rank test (*P* = 0.041, *P* = 0.048) (Figures 2 and 3). Further statistical analysis showed significant difference in clinical outcome among the patients treated with different chemotherapy strategies (*P* = 0.027).

### 3.3. Acute and Late Toxicity 

#### 3.3.1. Acute Toxicity

All patients tolerated the entire treatment. The main manifestations of acute toxicity were acute toxic reactions in the salivary glands, oral mucosa, and skin, and their G1, G2, and G3 incidence rates were 41.0%, 52.9%, and 6.1% for the salivary glands; 21.4%, 43.6%, and 35.0% for the oral mucosa, especially dysphagia requiring tube feeding (10.5%); and 72.3%, 24.0%, and 3.7% for the skin, respectively (see Table 4).

#### 3.3.2. Late Toxicity

**Figure 4:**
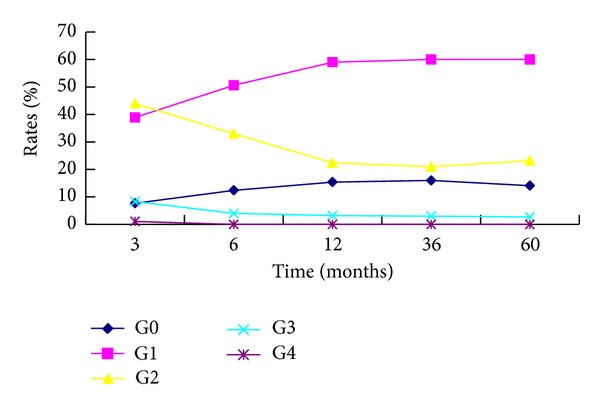
Diagram of correlative analysis between xerostomia and follow-up time.

The incidence rate for grade 3 late toxicity was 4.24%, including 2 cases of severe skin atrophy, 1 case of plate-like fibrosis in the skin of the neck, 19 cases of severe xerostomia, and 5 cases of trismus (the distance between upper and lower incisors < 1 cm) (see Table 5). The incidence rates of grades 0, 1, 2, 3, and 4 xerostomia were 7.7%, 38.9%, 44.0%, 8.4%, and 1.1% at 3 months; 15.4%, 59.0%, 22.4 %, 2.5%, and 0.0% at 1 year; and 14.1%, 60.0%, 23.2%, 2.7%, and 0.0% at 5 years after treatment, respectively. Xerostomia could be relieved to the greatest extent 3 to 6 months after treatment. However, it showed very little change after 1 year (see Figure 4).

### 3.4. Prognostic Factors

#### 3.4.1. Univariate Analysis

The impact of various prognostic factors on clinical outcomes was examined by univariate analysis. The LC of the patients with radiotherapy interruptions of ≤10 days and pretreatment hemoglobin levels of ≥110 g/L was higher than that of the patients with radiotherapy interruptions of >10 days and pretreatment hemoglobin levels less than 110 g/L. The T stage, N stage, clinical stage, radiotherapy interruption time, and pretreatment hemoglobin levels showed statistically significant impacts on the DMFS, DFS, and DSS rates, whereas T stage, N stage, clinical stage, radiotherapy interruption time, pretreatment hemoglobin level, extent of posttreatment weight loss, the use of chemotherapy, and gender all had effects on the OS rate (see Table 6).

#### 3.4.2. Multivariate Analysis

The independent risk factors that affected the local control rate included the radiotherapy interruption and T stage. The independent risk factors that affected the overall survival rate included N stage, use of chemotherapy, primary tumor volume, radiotherapy interruption, hemoglobin levels, and extent of weight loss (see Table 7).

The 5-year LC rate of the patients with nasopharyngeal gross tumor volumes (GTVnx) of ≤30 cm^3^ was significantly higher than that of the patients with GTVnx values of >30 cm^3^ (93.6% versus 80.4%, *P* < 0.05). The 5-year RC rate of the patients with GTVln values of ≤10 cm^3^ was higher than that of the patients with GTVln values of >10 cm^3^ (96.3% versus 85.0%, *P* < 0.05) (see Table 7).

## 4. Discussion

Radiotherapy is a primary therapeutic approach for the treatment of nasopharyngeal carcinoma. The transition from 2D-RT to three-dimensional RT (3DRT), in particular IMRT, represents a major step forward in the treatment of NPC. Although there are a number of dosimetric studies showing the advantage of IMRT over 2D-RT in treatment of NPC [1–3, 16], further clinical data are still needed. Lee et al. [6] reported the IMRT treatment results of 67 cases of untreated nasopharyngeal carcinoma (70% stage III/IV); it was indeed very encouraging, with an impressive 97% local PFS and 88% overall survival at 4 years when 70 Gy and 60 Gy were delivered to the GTV and PTV, respectively. Ng et al. [12] recently reported their IMRT experience in 193 NPC patients (93% stage III/IV disease) in Pamela Youde Nethersole Eastern Hospital (PYNEH) at Hong Kong. The 2-year local progression-free, regional progression-free, distant metastasis-free, and overall survival rates were 95%, 96%, 90%, and 92%, respectively. In this study, we analyzed the survival rates of 695 patients with primary nasopharyngeal carcinoma who were treated with IMRT at our center and found that the 5-year LC, RC, DMFS, DFS, DSS, and OS rates were 89.8%, 95.2%, 74.1%, 69.6%, 83.2%, and 77.1%, respectively. These results are similar to those obtained at other cancer centers. As reported previously, with NPC treated with 2D-RT alone, the 5-year overall survival was in the order of 50–70% for all stages [17–20].

Despite the recognized radiocurability and evidence of a dose-response relationship for NPC [21–24], radiotherapy treatment planning remains a great challenge in view of the close proximity of the tumor to the surrounding neural organs. In this study, the IMRT treatment plans were designed and optimized using the CORVUS treatment planning system. The average doses for *D*_01_ of the major serial organs, namely, the brain stem, spinal cord, optic chiasma, and left and right optic nerves, were 49.5 Gy, 37.4 Gy, 45.8 Gy, 42.8 Gy, and 43.9 Gy, respectively. The average doses for the parallel organs, namely, the left and right parotid glands and the left and right temporomandibular joints (*D*_33_), were 31.7 Gy, 32.1 Gy, 40.4 Gy, and 39.1 Gy, respectively. The radiation doses for each at-risk organ were significantly lower than their tolerance doses.

Distant metastasis is still the primary cause of treatment failure. Our follow-up results revealed 126 cases of treatment failure, including 29 cases of local failure, 13 cases of regional failure, and 117 cases of distant metastasis; thus, distant metastasis accounted for 73.6% of the cases of treatment failure. Of the 133 patients who died, 95 had distant metastasis, with bone metastasis being the most frequent site (47 cases). Zhao et al. [25] studied the effectiveness of IMRT-only treatment in 122 cases of nasopharyngeal carcinoma; out of the 17 cases of treatment failure that they observed, 70.6% were characterized by distant metastasis. Lin et al. [26] reported 230 nasopharyngeal carcinoma patients treated with IMRT, 5 cases of relapse in the primary site, 2 cases of relapse in the neck, and 16 cases of distant metastasis.

With the current IMRT treatment for NPC, we did not see any significant neurologic complications such as visual impairment, bulbar palsy, or symptomatic temporal lobe necrosis, which is expected to occur in a certain percentage of patients with extensive skull base disease if treated by conventional 2D-RT. In a study of 934 nasopharyngeal carcinoma patients treated with two-dimensional radiotherapy alone, Chen et al. [17] reported that the 5-year incidence rates for radiation-induced brain injuries, trismus, hearing loss, and xerostomia were 1.5%, 13.6%, 31.1%, and 38.7%, respectively. In contrast, our data show that the incidence rates for radiation-induced brain injuries and trismus were only 0.8% and 1.1%, respectively. Xerostomia was the most prominent late adverse effect, which could be relieved to the lowest degree within 3–6 months after the treatment, and the conditions did not show significant changes after 1 year posttreatment. At 6 months after IMRT treatment, G1, G2, and G3 xerostomia symptoms were found in 50.6%, 33.0%, and 4.0% of the patients, respectively; these values are somewhat lower than the 50%, 38.4%, and 9.6% reported by Zhang [27] for G1, G2, and G3 xerostomia, respectively. This may be due to the lower average radiation doses, 31.7 Gy for the left parotid gland and 32.1 Gy for the right parotid gland, received by the patients in this study. Nancy et al. [28] revealed that the rates of G1 and G2 xerostomia in nasopharyngeal carcinoma patients 3 months after IMRT treatment were 35% and 65%, respectively; 12 months after the treatment, these rates changed to 50% and 0%, respectively. Therefore, attention should be paid to the impact of follow-up time when adverse effects are assessed. Eisbruch et al. [29] demonstrated that the secretory function of the parotid gland was well preserved and gradually improved over time when the median dose used for its treatment was no greater than 26 Gy.

The synergistic and sensitizing effects of chemotherapy and radiotherapy provide a definite theoretical basis for improved therapeutic effects in the treatment of local advanced nasopharyngeal carcinoma through combined chemoradiotherapy. The use of the Intergroup regimen was shown to improve survival for patients with advanced NPC in the Intergroup 0099 trial and other series [30, 31]. However, it is less widely adopted in Asia because of concerns about the exact magnitude of benefit with this regimen in Asian patients. The situation has become better defined after the publication of the preliminary results of two randomized trials from Asia adopting the Intergroup 0099 regimen [32, 33]. In the randomized trial conducted by the Singapore group, 221 eligible patients with stages III and IV NPC were randomly assigned to RT alone or RT with concurrent and adjuvant chemotherapy. All patients had World Health Organization (WHO) type 2 NPC. The total scheduled dose of cisplatin was the same as the Intergroup regimen, but the method of administration of cisplatin was modified. Infusion of cisplatin was given in 4 days instead of infusion in 1 day. RT was given to 70 Gy in 35 fractions in 7 weeks. Chemoradiotherapy achieved significant improvement for distant control (2-year cumulative incidence rate for distant metastases, 16% versus 35%; *P* = 0.002) and overall survival (3-year actuarial rate, 80% versus 65%; *P* = 0.02). In another randomized trial conducted by the Hong Kong Nasopharyngeal Cancer Study Group (NPC9901), 348 patients with nonkeratinizing NPC staged T1-4N2-3M0 were randomly assigned to RT alone or RT with concurrent and adjuvant chemotherapy [34, 35]. At a median follow-up time of 6.1 years, adding chemotherapy statistically significantly improved the 5-year FFR (CRT versus RT: 67% versus 55%; *P* = 0.014) and 5-year progression-free survival (CRT versus RT: 62% versus 53%; *P* = 0.035). Cumulative incidence of acute toxicity increased with chemotherapy by 30% (CRT versus RT: 83% versus 53%; *P* < 0.001), but the 5-year late toxicity rate did not increase statistically significantly (CRT versus RT: 30% versus 24%; *P* = 0.30). Deaths because of disease progression were reduced statistically significantly by 14% (CRT versus RT: 38% versus 24%; *P* = 0.008), but 5-year overall survival was similar (CRT versus RT: 68% versus 64%; *P* = 0.22) because deaths due to toxicity or incidental causes increased by 7% (CRT versus RT: 1.7% versus 0 and 8.1% versus 3.4%, resp.; *P* = 0.015). Similarly, our study found that the 5-year distant metastasis-free survival and 5-year overall survival rate were 62.1% and 72.9%, respectively, for the IMRT-only treatment group and 70.9% and 79.1%, respectively, for the IMRT combined chemotherapy group, indicating that the use of combined chemotherapy significantly reduced the distant metastasis rate and improved the overall survival rate. Effective, less toxic chemotherapy agents are eagerly awaited to further improve the outcomes with lesser toxicity. Future studies should concentrate on the optimal sequencing of chemotherapy with RT.

We also conducted univariate and multivariate analyses of possible prognostic factors. Univariate prognostic analysis revealed that the OS rate was affected by gender, T/N stage, clinical stage, radiotherapy interruption, hemoglobin level, weight loss extent, and use of chemotherapy. However, multivariate analysis indicated that N stage, use of chemotherapy, and primary tumor volume were the most critical independent risk factors that affected the OS rate. The 5-year OS rate of the patients at stage III/IV was significantly lower than that of the patients at stage I/II, a result that is consistent with those reported in other studies. However, the T stage was not a prognostic factor that affected the OS rate; this is possibly because IMRT overcomes the disadvantages of conventional radiotherapy relating to dose distribution and further improves the conformality of target volume, leading to sufficient irradiation of the tumor target regions and improved survival rates of patients. The N stage was discovered to be another very important factor related to the OS rate in our study. The prognostic factor analysis of Han et al. [36], based on 305 patients with nasopharyngeal carcinoma treated with IMRT, showed that the T stage did not affect the LC and OS rates, whereas the N stage was an independent prognostic factor with an impact on OS. Specifically, the later the N stage is, the worse the prognosis is. The multivariate analysis of 122 patients with nasopharyngeal carcinoma treated with IMRT performed by Zhao et al. [25] also indicated that the N stage was an independent prognostic factor of DSS.

Due to the relationship between the tumor volume and biological side effects, tumor volume is considered to be a meaningful predictive factor. Chen et al. [37] found that the primary tumor volume was an independent prognostic factor of the local control rate and that the primary tumor volume was of more predictive significance than the T stage. Our study revealed that the 5-year LC rate of the patients with nasopharyngeal gross tumor volumes (GTVnx) of ≤30 cm^3^ was significantly higher than that of the patients with GTVnx values of >30 cm^3^. We also found that the 5-year RC rate of the patients with GTVln values of ≤10 cm^3^ was higher than that of the patients with GTVln values of >10 cm^3^. Lee et al. [38] conducted a retrospective analysis of 110 patients with NPC at stage III/IV, finding that large primary tumor volume and retropharyngeal lymph node volume GTVprn (≥13 mL) were significantly correlated with poor LC, DMFS, and prognosis. However, the accurate measurement of the tumor volume remains an important issue that requires further study.

Radiotherapy interruption time, anemia, and weight loss can also affect the prognosis. Wu and Zhao [39] reported that the DMFS rate significantly decreased when the radiotherapy interruption time was ≥5 days. However, our results showed that the DMFS rate only exhibited a statistically significant difference when the radiotherapy interruption time was >10 days, a result that is likely related to the higher total dose and higher single dose in the target area. This result suggests that the effects of radiotherapy interruption can be overcome by delivering an increased single dose and an increased total dose in the target area in those patients who experienced longer radiotherapy interruptions; however, this hypothesis needs to be validated with further evidence. The 5-year OS rate of the patients with normal pretreatment hemoglobin levels was higher than that of the patients with anemia (78% versus 70%). The 5-year OS rate of the patients with a body weight loss of less than 10% was higher than that of the patients with more than 10% body weight loss. These results are similar to those obtained by Huang et al. [40] and Hu [41] and provide a basis for further studies.

Although the current outcome of NPC treated with IMRT is encouraging, several questions still need to be solved. First, 15–20% incidence of distant metastasis is too high to be ignored, and more powerful and less toxic systemic therapy is required urgently to address this issue, such as molecular targeted therapy. Secondly, standardization on the method of delineation of GTV, CTV, and elective lymphatics region on CT should be worked out for a “common language” for cross-center communication. With the high conformity and steep dose gradient of IMRT, the risk of geographic miss is of special concern, and variation in the delineation criteria among radiation oncologists would make interpretation of pattern of local failure very difficult. Therefore, maximizing the local control and minimizing the risk of distant metastasis and late complications should be the key objectives in designing future clinical trials.

## Figures and Tables

**Table 1:** Clinical characteristics of 695 NPC.

Characteristics	*N* (%)
Age	
Median: 45 yrs	
Range: 14–74	
≤45 yrs	449 (64.6)
>45 yrs	246 (35.4)
Gender	
Male	540 (77.7)
Female	155 (22.3)
Histopathology (WHO type)	
Type 1	40 (5.7)
Types 2.1 & 2.2	655 (94.3)
T-classification	
T1	43 (6.1)
T2	229 (32.9)
T3	244 (35.1)
T4	179 (25.9)
N-classification	
N0	49 (7.1)
N1	228 (32.8)
N2	383 (55.1)
N3	35 (5.0)
Clinical stage	
I	43 (6.1)
II	172 (24.7)
III	268 (38.6)
IV	212 (30.6)
Chemotherapy	
Not given	236 (34.0)
Concurrent	190 (27.3)
Induction + concurrent	52 (7.5)
Concurrent + adjuvant	217 (31.2)

**Table 2:** Dose-volume statistics for targets (average result).

	*D*_max⁡_	*D*_min⁡_	*D*_mean_	Fractional dose	*V*_95_ (%)
GTVnx	78.24	66.37	72.62	2.25	99.4
GTVln-L/R	71.43	60.59	68.14	2.10	99.6
CTV1	68.77	61.27	65.21	1.94	99.4
CTV2	64.36	55.96	58.87	1.88	99.3
CTVln	54.19	50.04	51.42	1.82	99.3

GTVnx: gross target volume; GTVln-L/R: positive neck lymph nodes; CTV1: high-risk region of clinical tumor volume; CTV2: low- risk region of clinical tumor volume; CTVln: lymph node region of clinical tumor volume; *V*_95_: percentage of volume receiving 95% of prescribed dose.

**Table 3:** Dose-volume statistics for organs at risk.

OAR	Parameters	Mean (range)
Brain stem	*D*_01_	49.5 (45.6–54.7)
Spinal cord	*D*_01_	37.4 (32.1–45.5)
Optical chiasm	*D*_01_	45.8 (30.3–53.6)
Optical nerves		
L	*D*_01_	42.8 (26.9–48.9)
R	*D*_01_	43.9 (33.6–53.5)
Parotid gland		
L	*D*_33_	31.7 (20.2–33.9)
R	*D*_33_	32.1 (23.5–35.7)
Temporomandibular joints		
L	*D*_33_	40.4 (37.5–45.5)
R	*D*_33_	39.1 (30.8–42.8)

OAR: organs at risk; *D*_max⁡_: maximum point dose; *D*_01_: maximum dose to 1% volume.

**Table 4:** Frequency (%) of acute toxicity (Radiation Therapy Oncology Group acute radiation morbidity scoring criteria).

	Grade				
	0	1	2	3	4
Xerostomia	—	285 (41.0%)	368 (52.9%)	42 (6.1%)	—
Skin reaction	—	502 (72.3%)	167 (24.0%)	26 (3.7%)	—
Mucositis	—	149 (21.4%)	303 (43.6%)	243 (35.0%)	—
Dysphagia	—	284 (40.9%)	338 (48.6%)	73 (10.5%)	—

**Table 5:** Late toxic effects of radiotherapy.

	Content			
	G1 (%)	G2 (%)	G3 (%)	G4 (%)
Xerostomia	417 (60.0)	161 (23.2)	19 (2.7)	—
Hearing loss	20 (2.9)	2 (0.28)	—	—
Hypopsia	5 (0.7)	2 (0.28)	—	—
Brain injury	2 (0.28)	2 (0.28)	1 (0.14)	—

**Table 6:** Univariate prognostic analysis.

Prognostic factors	LC		DFS		DSS		OS	
	%	*P*	%	*P*	%	*P*	%	*P*
Gender								
Male	89.6	0.176	74.6	0.133	83.7	0.096	76.2	0.041
Female	91.0		72.1		81.1		78.7	
Age (years)								
≦45	92.4	0.853	75.5	0.737	84.4	0.589	78.8	0.745
>45	87.1		72.5		81.8		75.4	
T								
T1/2	91.5	0.887	85.7	<0.000	92.8	<0.001	83.0	0.012
T3/4	88.9		66.8		77.2		73.5	
N								
N0/1	93.5	0.718	80.6	0.004	90.5	<0.001	82.3	0.021
N2/3	88.6		70.5		78.3		73.7	
Clinical stage								
I + II	93.5	0.123	79.5	0.036	91.5	0.008	89.5	<0.001
III + IV	88.0		71.2		78.8		70.7	
Radiotherapy interruption								
≦10 d	92.6	0.000	75.6	0.000	84.9	0.000	79.4	0.000
>10 d	76.3		64.1		71.8		62.8	
HGB (g/L)								
≦110	77.0	0.000	63.3	0.000	73.3	<0.001	70.0	<0.001
>110	91.4		75.3		84.3		78.0	
Percentage of weight loss								
≦10%	90.9	0.092	75.6	0.525	86.5	0.153	80.5	0.011
>10%	85.6		72.4		79.6		73.5	
Chemotherapy or not given								
IMRT	88.9	0.385	72.2	0.204	81.8	0.087	72.7	0.043
Chemo. + IMRT	90.4		75.0		83.9		79.4	

HGB: hemoglobin; LC: local-regional control rates; DFS: disease-free survival; DSS: disease specific survival; OS: overall survival rate.

**Table 7:** Multivariate prognostic analysis.

Prognostic factors	LC		OS	
	HR	*P*	HR	*P*
T stage	4.592	<0.001	0.955	0.061
N stage	0.662	0.098	5.258	<0.001
GTVnx volume	1.825	0.064	3.846	<0.001
Radiotherapy interruption	5.481	<0.001	4.233	<0.001
HGB	0.339	0.463	1.905	0.002
Percentage of weight loss	0.229	0.621	1.018	0.033
Chemo. or not	0.683	0.221	4.353	<0.001

HGB: hemoglobin; LC: local-regional control rates; OS: overall survival rate; HR: hazards ratio.
